# Spontaneous haemothorax as a complication of Kassabach‐Merritt phenomenon (KMP) in a‐2‐years old female child: A case report

**DOI:** 10.1002/rcr2.1206

**Published:** 2023-09-04

**Authors:** Citra Cesilia, Elmi Ridar, Hariadi Hatta, Andreas Makmur, FW Wiwit Ade, Heda Melinda Nataprawira

**Affiliations:** ^1^ Department of Child Health, Arifin Achmad General Hospital University of Riau Pekanbaru Indonesia; ^2^ Department of Child Health, Division of Respirology, Hasan Sadikin General Hospital Universitas Padjadjaran Bandung Indonesia; ^3^ Department of Surgery, Arifin Achmad General Hospital University of Riau Pekanbaru Indonesia; ^4^ Department of Radiology, Arifin Achmad General Hospital University of Riau Pekanbaru Indonesia; ^5^ Department of Anathomical Pathology, Arifin Achmad General Hospital University of Riau Pekanbaru Indonesia

**Keywords:** children, corticosteroid, haemothorax, Kapossiform haemangioendothelioma, Kassabach‐Merritt Phenomenon

## Abstract

Kasabach‐Merritt phenomenon (KMP) is a rare condition that is associated with two rare vascular tumours: Kapossiform haemangioendothelioma (KHE) and tufted angioma (TA). A 2‐year‐old girl presented to our emergency room with a haemangioma and respiratory distress. The patient had a violaceous, palpable mass in the right upper chest since she was 5 months old. Severe anaemia, thrombocytopenia, coagulopathy, and hypofibrinogenemia were found. Chest x‐ray revealed massive pleural effusion in the right hemithorax. Chest computed tomography (CT) scanning revealed right pleural effusion, multiple destructions of bilateral ribs and multiple osteopenia of thoracic vertebrae. Chest CT angiography revealed a vascular mass in the sternum region. Based on clinical, laboratory and imaging findings, the diagnosis of KMP was established. Clinical, consumptive coagulopathy and thrombocytopenia were resolved by prednisone (3 mg/kg/day) and vincristine (1 mg/body surface area in m^2^/week) as an adjunct. Unfortunately, she had spontaneous rebleeding and died before the biopsy was done.

## Graphical abstract

Kasabach‐Merritt phenomenon (KMP) is a rare condition that is associated with two rare vascular tumours: Kapossiform haemangioendothelioma (KHE) and tufted angioma (TA). We present the fatal case of a 2‐years girl diagnosed with KMP based on clinical, laboratory and imaging findings but died due to spontaneous rebleeding before biopsy could be performed.

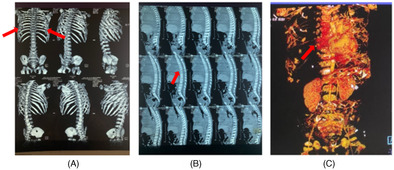

## INTRODUCTION

The Kasabach‐Merritt phenomenon (KMP) is characterized by a severe decrease in platelet count, a coagulation disorder leading to excessive blood clotting, and low fibrinogen levels. These symptoms are observed in connection with vascular tumours, namely Kapossiform haemangioendothelioma (KHE) or tufted angioma (TA).^1^ KMP exclusively applies to KHE, which manifests in 70% of instances.^2^ The increased depth and infiltration of the vascular tumour, along with its retroperitoneal or intrathoracic involvement, greatly magnify the peril associated with KMP.^3^ About 90% of all babies with KMP are diagnosed before 1 year. Children diagnosed with KMP are often susceptible to morbidity and mortality due to congenital vascular anomalies that result in haemorrhaging, invasion, or compression of critical structures by vascular tumours. Such occurrences are commonly observed in conjunction with children affected by KMP.^4^

## CASE REPORT

**FIGURE 1:**
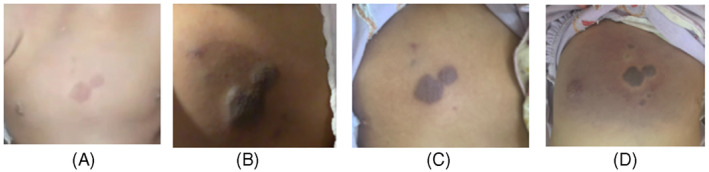
The progression of the patient's haemangioma. (A) at 5 months of age, (B) At first hospital admission at 2 years old of age, when KMS diagnosis was established, (C) after initial steroid therapy and (D) during rebleeding.

A 2‐year‐old girl presented with gradually progressing shortness of breath and pain after she fell 2 months previously. She had a violaceous, palpable mass in the right upper chest, which was suggested as a birthmark, since she was 5 months old (Figure 1). She looked moderately ill on physical examination, fully conscious, and tachypnoeic. On physical examination of the chest, there was a retraction, a protruding right hemithorax and lagging right thoracic movements.

**FIGURE 2:**
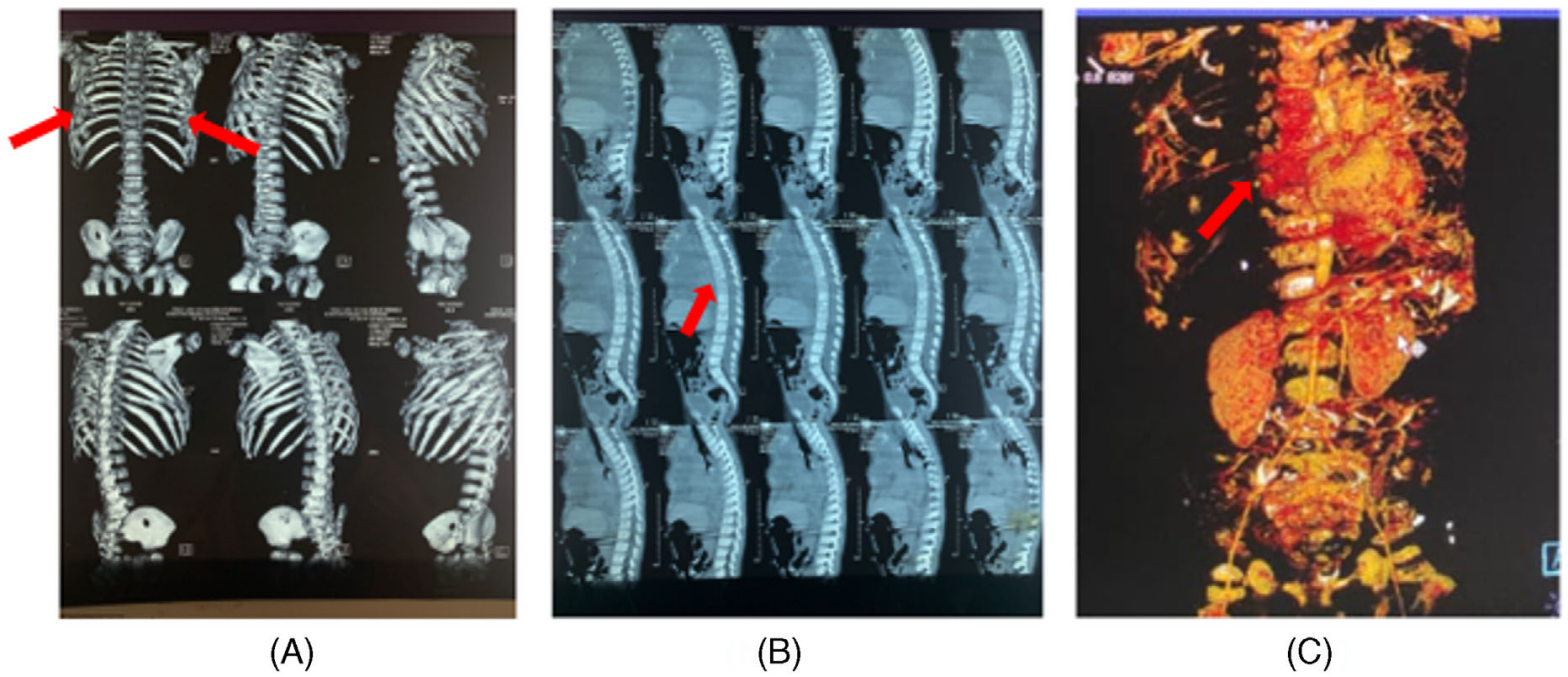
(A) Chest 3D CT‐angiography revealed multiple rib destructions of rib 9, 8, 5, 4, 3, 2 and 1 anterior dextra and rib 4, 3, 2 and 1 anterior sinistra, (B) multiple osteopenia of thoracic vertebrae and (C) vascular mass in the sternum region.

Laboratory examination showed anaemia (Haemoglobin 6.8 g/dL), thrombocytopenia (platelets 26.000/mm^3^), prothrombin time (PT) prolongation, increased international normalized ratio (INR 1.48) and D‐Dimer (13.8 pg/mL), and hypofibrinogenaemia (Fibrinogen 95 mg/dL). The chest x‐ray showed a massive right pleural effusion. We performed pleural tapping with ultrasound guiding and obtained 30 mL of blood with the results of exudate analysis and histopathological examination showing atypical cells without malignant cells. Chest CT scan showed right pleural effusion, destruction of multiple bilaterally anterior ribs, and suspected osteopenia in multiple vertebrae. Chest CT‐angiography showed a vascular mass in the cutis sternal region (Figure 2). We performed thyroid stimulating hormone (TSH) sensitive and Free T4, parathyroid hormone, calcium, phosphorus, and 25‐OH levels tests to determine the aetiology of osteopenia in the patient, but all results were within normal limits. She was treated with prednisone (3 mg/kg body weight/day) and vincristine (1 mg/ body surface area in m^2^/week). However, a few days after starting vincristine, she died due to spontaneous rebleeding before we performed the biopsy. Based on KMP findings and avascular necrosis bone destruction in multiple costae and vertebrae, we highly suspect the vascular tumour is a KHE or TA with complications of KMP.

## DISCUSSION

Standard diagnostic criteria for the diagnosis of KMP have yet to be established.^5^ Generally, confirmation of the diagnosis of KMP requires a complete blood count (for thrombocytopenia, hypofibrinogenemia and increased D‐dimer) to confirm consumptive coagulopathy and radiological examinations, usually using MRI and CT‐scans, to confirm the diagnosis of other vascular tumours that can be found together in patients with KMP.^1^, ^2^, ^5^ KMP is associated with vascular tumours such as KHE, with a study reporting 70% of KHE cases had previous diagnosis of KMP.^2^ In infants, KHE is an uncommon vascular neoplasm.^2^, ^5^ The extremities, trunk, and retroperitoneum are typically affected by a solitary, enlarging lesion with a firm texture that displays purpuric characteristics in both the cutaneous and soft tissue.^2^ The confirmation of various vascular tumour types, which may result in KMP‐related clinical manifestations, can be achieved through biopsy examinations.^1^, ^3^, ^5^ Despite biopsy being the gold standard for diagnosing vascular tumours, it is often not feasible in cases of severe KMP in KHE due to the potential worsening of coagulopathy and increased bleeding risk.^5^ Biopsy specimens should be considered to ascertain the diagnosis in case‐by‐case basis, particularly with atypical clinical manifestation.^5^

The primary objectives of treatment of paediatric patients affected by KMS are to effectively manage coagulopathy and maximizing the rate of regression observed in hemangiomas.^5^ First‐line therapy for KMP is typically the administration of systemic corticosteroids, effective for restoring platelet counts to normal levels.^5^ Recent finding have indicated that corticosteroids may be utilized in conjunction with other treatments for KMP.^1^, ^5^ Consensus‐derived guidelines recommend vincristine monotherapy or vincristine plus corticosteroids as the primary treatment for cases of KHE with KMP.^5^ After the administration of prednisone and vincristine, our patient had exhibited clinical improvement as well as laboratory results indicating increased platelet count and resolution of consumptive coagulopathy. Unfortunately, spontaneous rebleeding occurred a few days after vincristine administration before the biopsy was done. Bleeding due to vascular tumour rupture and invasion or compression of vital structures from vascular tumours, a fatal complication of KMP with KHE, is frequently found together.^1^ The cause of spontaneous rebleeding in this case may be associated with severe rib destruction that may potentially compress the vascular tumours, leading to their rupture and subsequent death due to haemorrhage potentially from the tumour rupture.

In conclusion, KMP is a potentially severe and rare complication in children and might contribute to significant risk of morbidity and mortality. Therefore, timely diagnosis and effective treatment are crucial in enhancing the patient's long‐term prognosis. CT scans and angiography are effective in achieving greater diagnostic accuracy for KMP. Although biopsy is the gold standard for diagnosis, it should be performed on a case‐by‐case basis. Despite its relatively low response rate and tolerance, prednisone is commonly employed as the initial treatment option, while vincristine is used as an additional therapy. Rebleeding is a potentially leading cause of death in children with KMP.

## AUTHOR CONTRIBUTIONS

Citra Cesilia drafted the manuscript under the supervision of Heda Melinda Nataprawira. Citra C, Elmi, Hariadi H, Andreas M and FW Wiwit Ade were expert team for this case. All authors critically revised the manuscript for important intellectual content and approved the final version of the manuscript before submission for publication.

## CONFLICT OF INTEREST STATEMENT

None declared.

## ETHICS STATEMENT

The authors declare that appropriate written informed consent was obtained for the publication of this manuscript and accompanying images.

## Data Availability

The data that support the findings of this study are available on request from the corresponding author. The data are not publicly available due to privacy or ethical restrictions.
